# Supplementary material for: Association of the Metabolic Score Using Baseline FDG-PET/CT and dNLR with Immunotherapy Outcomes in Advanced NSCLC Patients Treated with First-Line Pembrolizumab
Source: Cancers (Basel). 2020 Aug 10;12(8):2234. doi: 10.3390/cancers12082234 (PMC7463532; doi:10.3390/cancers12082234)
Supplement: Supplementary file 1 [file cancers-12-02234-s001.pdf]

Supplementary Materials

Association of the Metabolic Score using Baseline FDG-PET/CT and dNLR with Immunotherapy Outcomes in Advanced NSCLC Patients Treated with First-Line Pembrolizumab.

Romain-David Seban, Jean-Baptiste Assié, Etienne Giroux-Leprieur, Marie-Ange Massiani, Michael Soussan, Gérald Bonardel, Christos Chouaid, Margot Playe, Lucas Goldfarb, Boris Duchemann, Laura Mezquita, Nicolas Girard and Laurence Champion

Material and Methods

Acquisition parameters for each PET/CT device:

The following four PET/CT scanners were used:

- General Electric Discovery-690 (GE Healthcare, Waukesha, WI) (15 patients) and GE Discovery-710 (7 patients), both with LYSO-based detectors,
- GE Discovery-MI (27 patients) and Philips PET/CT Vereos (14 patients), both combining the small LYSO with the SiPM block design (LightBurst digital detector).

The PET images were reconstructed using the following iterative algorithms:

- GE Discovery-690: Vue Point FX algorithm, time of flight (TOF) reconstruction, matrix 256 × 256, 2 iterations, 24 subsets, post-filter 6.4mm;
- GE Discovery-710: Vue Point HD algorithm, time of flight (TOF) reconstruction, matrix 256 × 256, 2 iterations, 12 subsets, post-filter 6.4mm;
- GE Discovery-MI: Vue Point FX algorithm, time of flight (TOF) reconstruction, matrix 256 × 256, 2 iterations, 17 subsets, post-filter 6.4mm;
- Philips Vereos: OSEM algorithm, time of flight (TOF) reconstruction, matrix 288 × 288, 3 iterations, 5 subsets, post-filter 2mm.

Definition of outcomes:

- Overall Survival (**OS**): defined as the time from the first ICI perfusion to the date of death, due to any cause, or to the date of censoring at the last time the patient was known to be alive.
- Progression-Free Survival (**PFS**): defined as the time from the first pembrolizumab perfusion to disease progression or death from any cause.
- Overall Response Rate (**ORR**): defined as the percentage of patients with a best overall response of complete response (CR) or partial response (PR) at any time during the treatment.
- Disease Control Rate (**DCR**): defined as the percentage of patients with a best overall response of complete response (CR), partial response (PR) or stable disease (SD) at any time during the treatment.
- Length of follow-up: calculated from the date of the initial PET/CT to the date of the last clinical consultation.

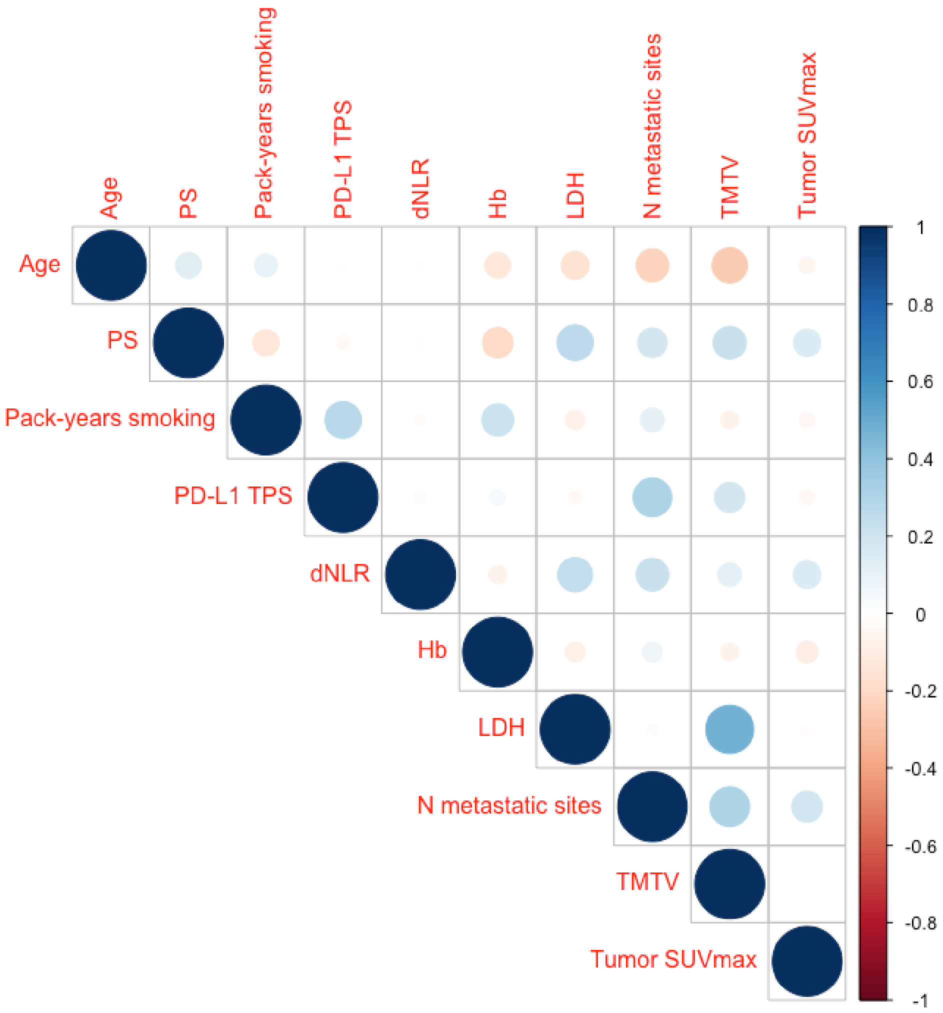

Figure S1. Correlations between tumor SUVmax, TMTV and clinical or biological variables (correlogram).

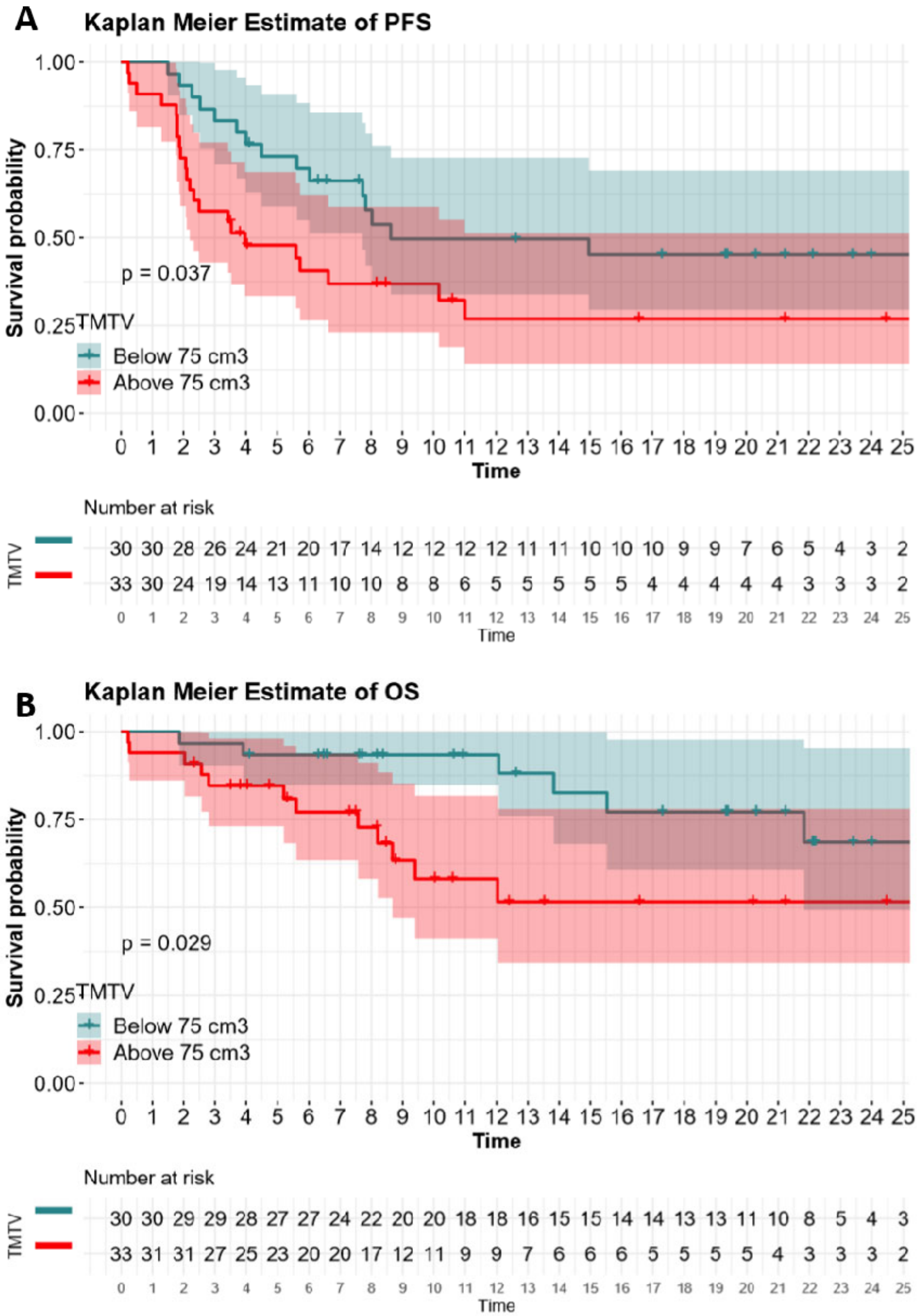

Figure S2. Kaplan-Meier curves for (**A**) Progression-Free Survival (PFS) and (**B**) Overall Survival (OS) stratified according to TMTV in all patients (*n* = 63).

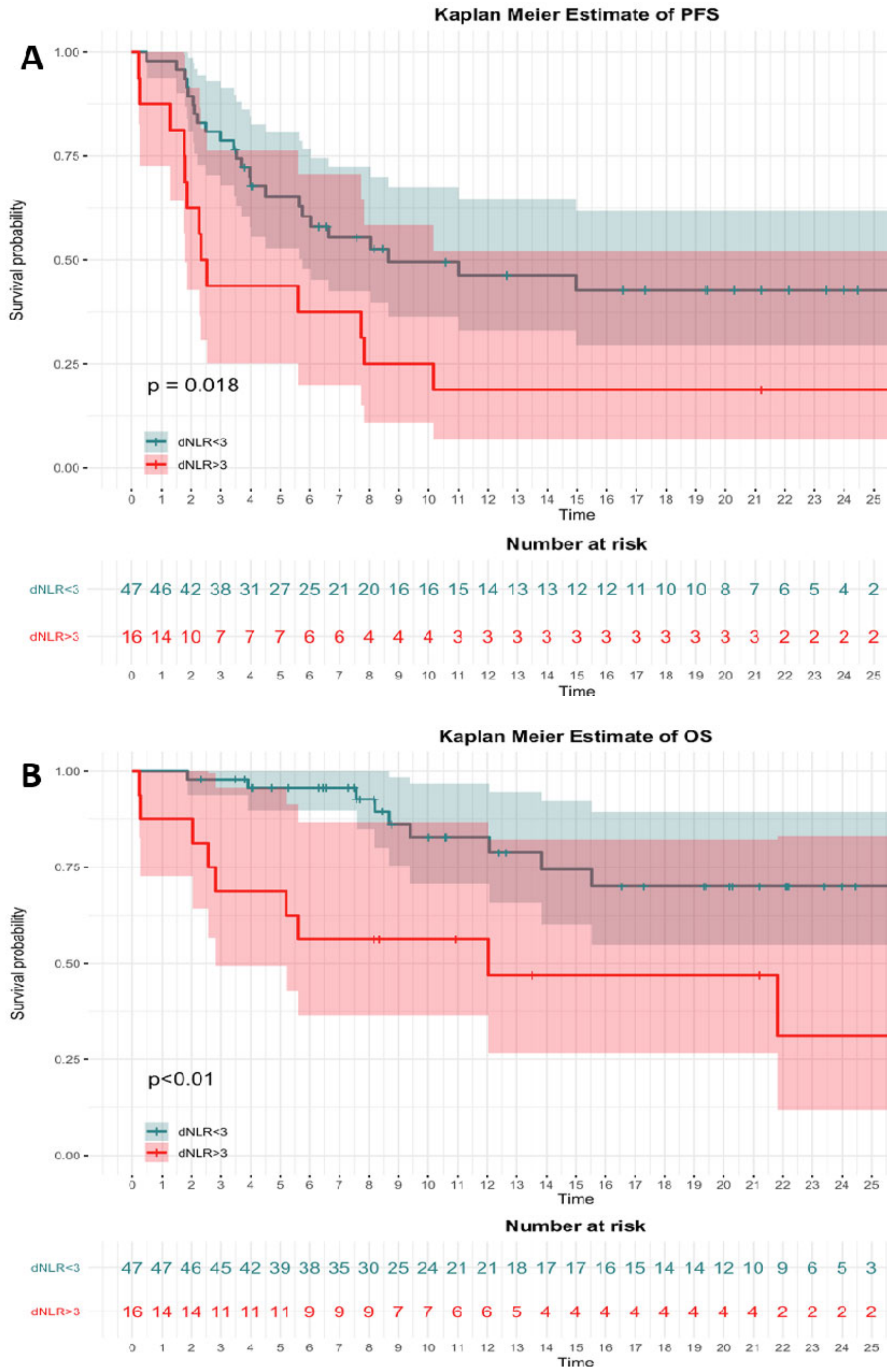

Figure S3. Kaplan-Meier curves for (**A**) Progression-Free Survival (PFS) and (**B**) Overall Survival (OS) stratified according to dNLR in all patients (*n* = 63).

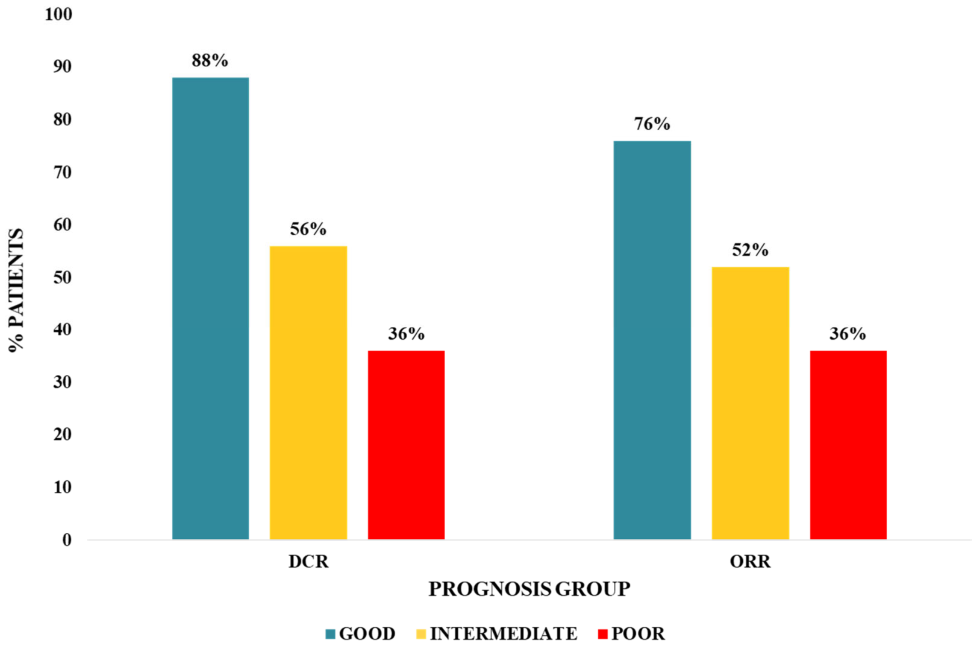

Figure S4. Disease control rate (DCR %) and overall response rate (ORR %) according to the metabolic score.

Table S1. Correlations between TMTV, tumor SUVmax and clinical or biological variables (Spearman's coefficient).

	**Correlation with Tumor SUVmax**		**Correlation with TMTV**	
	**Spearman's coefficient**	*p* value	Spearman's coefficient	*p* value
**Clinical parameters**				
Age	−0.12	0.32	**−0.29**	**0.02**
PS	0.19	0.10	0.23	0.06
Pack-years smoking*	0.01	0.96	−0.20	0.17
**PD-L1 expression**				
TPS (%)	0.14	0.28	0.22	0.08
**Biology**				
dNLR	0.18	0.13	0.10	0.46
Hemoglobin	−0.17	0.18	−0.17	0.18
LDH**	−0.01	0.92	**0.41**	**<0.01**
**FDG-PET parameters**				
Tumor SUVmax	-	-	0.03	0.81
TMTV	0.03	0.81	-	-

Note: * Only smokers (former or current: $n = 60$ pts) were included. ** Available data for a total of 40 patients (63%). Bold values denote statistical significance at the $p < 0.05$ level.

Table S2. Patients treated with corticosteroids at baseline: drug name, doses and indications.

Patient	Type of Corticosteroid	Daily Dose (mg Prednisone)	Reason for Corticosteroid Treatment
1	prednisolone	80	brain metastasis
2	prednisolone	30	corticotroph deficiency
3	prednisolone	80	brain metastases
4	prednisone	80	brain metastases
5	prednisone	80	colitis
6	prednisolone	60	epiduritis
7	prednisone + fluticasone (inhaled)	40 (oral) + 4 (inhaled)	dyspnea
8	prednisone	80	brain metastases
9	prednisolone	10	epiduritis
10	prednisolone	60	superior vena cava syndrome
11	prednisone	40	brain metastases
12	prednisone	20	brain metastases

Table S3. Comparison of the baseline characteristics of patients with or without corticosteroids at baseline.

	**Patients with Corticosteroids (*n* = 13 pts)**	**Patients without Corticosteroids (*n* = 50 pts)**	
Variable	median (min–max)	median (min–max)	*p* value
Age	65 (48–86)	65 (37–85)	0.83
Performance status (PS)	1 (0–2)	1 (0–3)	0.84
Pack-years smoking	30 (20–65)	35 (5–100)	0.23
PD-L1 TPS (%)	70 (50–100)	80 (50–100)	0.49
dNLR	2.4 (1.1–14.1)	2.1 (0.9–6.7)	0.16
Hemoglobin (g/dL)	12.8 (9.5–15.4)	12.6 (8.3–16.0)	0.70
LDH* (UI/L)	205 (160–2197)	259 (105–1651)	0.57
N metastatic sites	2 (0–6)	2 (0–5)	0.25
Tumor SUVmax	22.4 (10.4–31.6)	17.7 (5.4–41.4)	0.07
TMTV (cm^3)	94.1 (26.9–209.2)	83.7 (12.4–427.9)	0.94

Table S4. Comparison of the baseline characteristics of high or very-high PD-L1 expressors.

	**Patients with very high PD-L1 expression TPS: 90–100 % (*n* = 19 pts)**	**Patients with high PD-L1 expression TPS: 50–89 % (*n* = 44 pts)**	
Variable	median (min-max)	median (min–max)	p value
Age	67 (55–85)	63 (37–86)	0.26
Performance status (PS)	1 (0–3)	1 (0–3)	0.55
Pack-years smoking	40 (5–100)	31 (10–75)	0.08
PD-L1 TPS (%)	95 (90–100)	60 (50–80)	<0.01
dNLR	2.1 (0.8–14.1)	2.2 (1.0–8.6)	0.78
Hemoglobin (g/dL)	12.4 (9.3–15.6)	12.8 (8.3–16.0)	0.76
LDH* (UI/L)	254 (141–576)	232 (105–2197)	0.20
N metastatic sites	2 (0–5)	2 (1–6)	0.46
Tumor SUVmax	18.8 (5.9–28.0)	17.6 (5.4–41.4)	0.77
TMTV (cm^3)	96.2 (42.3–269.8)	69.2.7 (12.4–427.9)	0.07

Note: * Available data for a total of 40 patients (63%).

Table S5. Comparison of the baseline characteristics of patients with squamous or non-squamous carcinoma.

	**Squamous (*n* = 13 pts)**	**Non-squamous (*n* = 50 pts)**	
Variable	median (min–max)	median (min-max)	*p* value
Age	72 (54–86)	64 (37–85)	0.05
Performance status (PS)	1 (0–3)	1 (0–2)	0.08
Pack-years smoking	34 (10–100)	35 (5–90)	0.87
PD-L1 TPS (%)	80 (50–100)	80 (50–100)	0.83
dNLR	2.3 (0.9–6.4)	2.1 (1.0–14.1)	0.77
Hemoglobin (g/dL)	12.1 (8.3–14.7)	12.7 (8.8–16.0)	0.24
LDH* (UI/L)	198 (105–323)	259 (141–2197)	0.21
N metastatic sites	2 (0–4)	2 (0–6)	0.68
Tumor SUVmax	20.6 (5.7–41.4)	16.5 (5.4–31.8)	0.02
TMTV (cm^3)	84.5 (20.2–185.1)	82.7 (12.4–427.9)	0.41

Note: * Available data for a total of 40 patients (63%).

Table S6. Baseline patient characteristics in the three prognostic groups according to the metabolic score.

Metabolic Score<br>*n* (%)	Good Prognosis Group<br>25 (40%)	Intermediate Prognosis Group<br>27 (43%)	Poor Prognosis Group<br>11 (17%)
**ECOG PS**			
≥ 2	3 (12%)	5 (19%)	5 (45%)
**Histology**			
SCC	3 (12%)	8 (30%)	2 (18%)
**Smokers**			
Always	23 (92%)	26 (96%)	11 (100%)
**dNLR**			
>3	0 (0%)	5 (19%)	11 (100%)
**Hemoglobin**			
≤ 12 g/dL	4 (16%)	11 (41%)	7 (64%)
**LDH***			
> ULN	6 (24%)	8 (30%)	5 (45%)
**N metastatic sites**			
> 3	2 (1%)	5 (19%)	4 (36%)
**Liver metastasis**			
Yes	0 (0%)	5 (19%)	4 (36%)
**Tumor SUVmax**			
> 16.5	12 (48%)	5 (19%)	7 (64%)
**TMTV**			
> 75 cm^3	0 (0%)	22 (81%)	11 (100%)

Note: * Available data in a total of 40 patients (63%).

Table S7. Summary of published data for combination of blood inflammatory markers and FDG-PET parameters in advanced NSCLC.

Drug	Line of Therapy	Study	N Patients	Name	Blood Marker (Cut-Off if Categorial Variable)	FDG-PET Marker (Cut-Off if Categorial Variable)	Groups	Outcomes	Significance	Reference
Nivolumab Pembrolizumab Atezolizumab	First-line (16%) Second or further-line (84%)	Retrospective	80	Metabolic Score	dNLR (>3) At baseline	TMTV (>75cm3) At baseline	High risk: 2 factors Intermediate risk: 1 factor Low risk: 0 factor	High risk: poor PFS and OS + no DCB Low risk: good PFS and OS + DCB	**High risk** ($p < 0.001$): PFS: 1.4 months (95% CI 0.8–2.0) OS: 2.4 months (95% CI 1.9–2.9) **Low risk:** DCB: OR 7.9; $p = 0.003$	[1]
Nivolumab Pembrolizumab	First-line (24%) Second or further-line (76%)	Prospective	33	IMPI	NLR (>4.9) At first restaging	TLG (>541.5 ml) At first restaging	High risk: 2 factors Intermediate risk: 1 factor Low risk: 0 factor	High risk: poor PFS and OS Low risk: good PFS and OS	**High risk** ($p < 0.001$): PFS: 1.8 months (95% CI 1.6–2.0) OS: 2.8 months (95% CI 1.4–4.0)	[2]
Nivolumab Pembrolizumab Atezolizumab Nivolumab+Ipililumab	First-line (26%) Second or further-line (74%)	Prospective	46		dNLR (continuous variable) At baseline	TMTV (continuous variable) At baseline		OS Association with HPD	**TMTV** OS: HR, 1.0; $p = 0.008$ HPD: $p = 0.01$ **dNLR** OS: HR, 1.2; $p = 0.01$ HPD: $p = 0.04$	[3]

Abbreviations

BOR	best overall response
**CI**	confidence interval
**CT**	computed tomography
**DCB**	disease clinical benefit
**DCR**	disease control rate
**dNLR**	derived neutrophils-to-lymphocytes ratio
**ECOG**	eastern cooperative oncology group
**FDG**	fluoro-deoxy-glucose
**HD**	high definition
**HPD**	hyperprogressive disease
**HR**	hazard ratio
**ICIs**	immune checkpoint inhibitors
**LDH**	lactate dehydrogenase
**LYSO**	lutetium yttrium oxyorthosilicate
**NSCLC**	non-small cell lung cancer
**ORR**	overall response rate
**OS**	overall survival
**OSEM**	ordered subset expectation maximization
**PET**	positron emission tomography
**PD-L1**	programmed cell death ligand-1
**PFS**	progression-free survival
**SCC**	squamous cell carcinoma
**SiPM**	silicon photomultipliers
**SUVmax**	maximum standard uptake value
**TMTV**	total metabolic tumor volume
**TOF**	time of flight
**TPS**	tumor proportion score

References

1 Seban, R.-D.; Mezquita, L.; Berenbaum, A.; Dercle, L.; Botticella, A.; Le Pechoux, C.; Caramella, C.; Deutsch, E.; Grimaldi, S.; Adam, J.; et al. Baseline metabolic tumor burden on FDG PET/CT scans predicts outcome in advanced NSCLC patients treated with immune checkpoint inhibitors. *Eur. J. Nucl. Med. Mol. Imaging* **2020**, *47*, 1147–1157, doi:10.1007/s00259-019-04615-x.

2 Castello, A.; Toschi, L.; Rossi, S.; Mazziotti, E.; Lopci, E. The immune-metabolic-prognostic index and clinical outcomes in patients with non-small cell lung carcinoma under checkpoint inhibitors. *J. Cancer Res. Clin. Oncol.* **2020**, *146*, 1235–1243, doi:10.1007/s00432-020-03150-9.

3 Castello, A.; Rossi, S.; Toschi, L.; Mazziotti, E.; Lopci, E. Hyper-progressive Disease in Patients With Non-Small Cell Lung Cancer Treated With Checkpoint Inhibitors: The Role of 18F-FDG PET/CT. *J. Nucl. Med.* **2019**, doi:10.2967/jnumed.119.237768.

© 2020 by the authors. Licensee MDPI, Basel, Switzerland. This article is an open access article distributed under the terms and conditions of the Creative Commons Attribution (CC BY) license (http://creativecommons.org/licenses/by/4.0/).
